# Teaching of manual clinical skills in podiatry: theory and recommendations

**DOI:** 10.1186/1757-1146-6-S1-P1

**Published:** 2013-05-31

**Authors:** Ryan Causby, Susan Hillier, Michelle McDonnell, Lloyd Reed

**Affiliations:** 1 School of Health Sciences, University of South Australia, Adelaide, South Australia, 5074, Australia; 2 School of Clinical Sciences, Queensland University of Technology, Brisbane, Queensland, 4064, Australia

## 

It is the expectation of employers, regulatory bodies and the public, that graduating podiatrists sufficiently meet certain minimum competencies for that profession, including those for manual skills. However, teaching and evaluation methods seem to be inconsistent between countries, institutions and programs. This may be the consequence of uncertainty regarding the most effective method of teaching such skills.

A review of available literature pertaining to psychomotor teaching across a range of health professions was undertaken. As a result of this broad review we present the available evidence and make recommendations pertaining to the teaching of psychomotor skills within the podiatry profession and relate it to current methods.

Traditional methods of teaching providing explicit content and appropriate demonstration are still useful. Learning may be promoted in a closed environment on low fidelity models with clear and immediate feedback. Further practice can occur over time (intermittent practice) with possible use of mental practice in between. The task can gradually increase in complexity such as moving from a model to work in a clinical setting such as a university clinic on real patients, as appropriate. Further detail regarding these methods will be provided.

This review will support some current practices in clinical teaching and make further recommendations with respect to current evidence.

